# Phylogeography of *Pinus armandii* and Its Relatives: Heterogeneous Contributions of Geography and Climate Changes to the Genetic Differentiation and Diversification of Chinese White Pines

**DOI:** 10.1371/journal.pone.0085920

**Published:** 2014-01-21

**Authors:** Liu Liu, Zhen-Zhen Hao, Yan-Yan Liu, Xiao-Xin Wei, Yu-Zhi Cun, Xiao-Quan Wang

**Affiliations:** 1 State Key Laboratory of Systematic and Evolutionary Botany, Institute of Botany, Chinese Academy of Sciences, Beijing, China; 2 Graduate University of the Chinese Academy of Sciences, Beijing, China; 3 Dali University, Dali, Yunnan, China; BiK-F Biodiversity and Climate Research Center, Germany

## Abstract

Geographic barriers and Quaternary climate changes are two major forces driving the evolution, speciation, and genetic structuring of extant organisms. In this study, we used *Pinus armandii* and eleven other Asian white pines (subsection *Strobus*, subgenus *Pinus*) to explore the influences of geographic factors and Pleistocene climatic oscillations on species in South China, a region known to be centers of plant endemism and biodiversity hotspots. Range-wide patterns of genetic variation were investigated using chloroplast and mitochondrial DNA markers, with extensive sampling throughout the entire range of *P. armandii*. Both cpDNA and mtDNA revealed that *P. armandii* exhibits high levels of genetic diversity and significant population differentiation. Three geographically distinct subdivisions corresponding to the Qinling-Daba Mountains (QDM), Himalaya-Hengduan Mountains (HHM) and Yungui Plateau (YGP) were revealed in mainland China by cpDNA. Their break zone was located in the southeastern margin of the Qinghai-Tibetan Plateau (QTP). A series of massive mountains, induced by the QTP uplift, imposed significant geographic barriers to genetic exchange. The disjunct distribution patterns of ancestral haplotypes suggest that a large continuous population of the white pines may have existed from southwest to subtropical China. Repeated range shifts in response to the Pleistocene glaciations led to the isolation and diversification of the subtropical species. The two Taiwanese white pines share a common ancestor with the species in mainland China and obtain their chloroplasts via long-distance pollen dispersal from North Asian pines. Distinct genetic patterns were detected in populations from the Qinling-Daba Mountains, Yungui Plateau, Himalaya-Hengduan Mountains, and subtropical China, indicating significant contributions of geographic factors to the genetic differentiation in white pines. Our study depicts a clear picture of the evolutionary history of Chinese white pines and highlights the heterogeneous contributions of geography and Pleistocene climatic fluctuations to the extremely high plant species diversity and endemism in South China.

## Introduction

Geographic barriers and climate change are two major forces driving the evolution, speciation and genetic structuring of extant organisms [1]–[2]. The Cenozoic collision between India and Asia created a highly complex montane system in southwest China, including the highest mountain range on Earth (the Himalayas) and the largest “roof of the world”-the Qinghai-Tibet Plateau (QTP). The uplift of the QTP reshaped the landscape features of central and eastern Asia and the global climate system [3]–[6]. In conjunction with the dramatic cooling of the global climate in the past three million years, geologic changes induced by the recent uplift of the QTP would be expected to have contributed to the development of the extremely high levels of species diversity in South China, a large region known to be centers of plant endemism and biodiversity hotspots. In this vast mountainous area, the Hengduan Mountain range, Central China, and the Nanling Mountains, which span the three-tier terrain of China from the high elevation west to the low elevation east, were recognized as plant diversity and endemism hotspots [7]. In particular, the Himalaya-Hengduan Mountains (HHM) region, which extends along the southern frontier to the southeastern margin of the QTP, encompasses 2 of the 34 biodiversity hotspots of the world, i.e., ‘Mountains of Southwest China’ and ‘Himalaya’ [8]. Although there is widespread agreement that dramatic geomorphology changes induced by the QTP uplift and/or Pleistocene glaciations profoundly affected the biota in this region, their relative influences on speciation and genetic structuring are largely unknown.

The last decade has witnessed an exponential growth in phylogeographic studies on the diverse taxa of many geographic regions in China (e.g., [9]–[12]). Unfortunately, most studies sampled only a single or a few closely related species, and concentrated extensively on the effects of Quaternary glaciations on genetic structure and the locations of glacial refugia. Only recently, have a few studies begun to disentangle the relative influences of the uplift of the QTP and the Pleistocene climatic fluctuations on speciation and genetic structuring in the QTP region and adjacent areas [13]–[16]. Moreover, there are few large-scale phylogeographic studies with sufficient coverage of distinct bioregions (for instance, warm central China, subtropical southern China, and mountainous western China) to detect the diversity of responses among closely related species to the geologic changes induced by the uplift of the QTP and Pleistocene glaciations.

**Figure 1:**
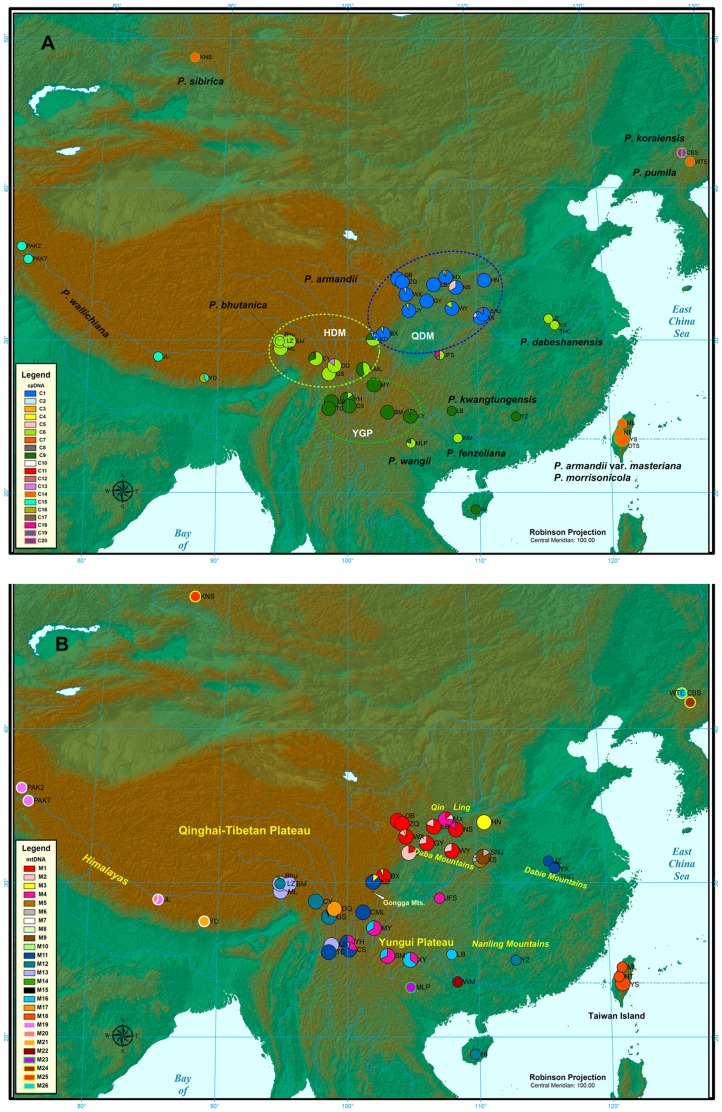
Geographic distribution of chlorotypes (A) and mitotypes (B). Small circles in the pie charts indicate populations from the relatives of *Pinus armandii*. The three geographic subdivisions of *P. armandii* in mainland China are shown in A.

The Chinese white pines, *Pinus armandii* and its relatives (*Pinus*, subgenus *Strobus*) provide an efficient system to explore these historical processes due to their extensive geographic distributions and relatively recent diversification (10∼20 million years ago; Ma) [17]. Although there is no universal agreement on taxonomic relationships, most studies, including our own recent molecular phylogenies (unpublished), show a close relationship among the nine white pines distributed in the south part of mainland China and Taiwan, i.e., *P. armandii* and its variety *P. armandii* var. *mastersiana*, *P. bhutanica*, *P. dabeshanensis*, *P. fenzeliana*, *P. kwantungensis*, *P. morrisonicola*, *P. wallichiana*, and *P. wangii* [18]–[22]. All of these white pines are assigned to the subsection *Strobus* of Section *Quinquefoliae* [23]. Among them, *P. armandii* has the broadest geographic distribution. It is extensively and discontinuously distributed in central and southwest China, across a latitudinal range of 23°30′–36°31′N and a longitudinal range of 85°30′–113°E, with one variety, *P. armandii* var. *mastersiana*, extending to Taiwan [24]. *P. wallichiana* is a common conifer tree occurring throughout the temperate regions of the Himalayas, whereas another Himalayan pine, *P. bhutanica*, is only endemic to the eastern Himalayas. Three very closely related pines, *P. fenzeliana*, *P. kwantungensis*, and *P. wangii*, have localized and patchy distributions in the lower mountains and montane forests of southern China and possibly in adjacent northern Vietnam. *P. dabeshanensis* is a rare pine endemic to the Dabie Mountains, where it represents the easternmost distribution of soft pines in mainland China. In North China, three widespread north Asian white pines, *P. sibirica*, *P. pumila* and *P. koraiensis*, have a relatively limited distribution in the northwest and northeast (Fig. 1A).

In this study, we used a combination of phylogeography, inter-specific phylogeny and molecular dating techniques with two paternally inherited chloroplast DNA fragments and three maternally inherited mitochondrial DNA markers to elucidate the effects of geography and Quaternary climatic oscillations on the genetic structure, speciation and evolution of the Chinese white pines. This study will shed further light on the mechanisms underlying plant speciation and the origin of the strikingly high plant diversity in southern China.

## Materials and Methods

### Ethics statement

No specific permits were required for the described locations in China because all researchers collecting the samples had introduction letters from the Institute of Botany, Chinese Academy of Sciences (IBCAS), Beijing. The samples from northern Pakistan were obtained from the international scientific research project of “Flora of Pan-Himalaya”. The field studies involved collection of endangered species.

### Population sampling

A total of 415 individuals from 30 populations of *P. armandii* and *P. armandii* var. *mastersiana*, representing the entire geographic range of this species, were included in our analysis. Each population had 13–23 individuals except for six from mainland China (DB, ZQ, LB, NS, SNJ, and HN) and two from Taiwan (DTS and YS), which were represented by <10 individuals. Sixty-five additional trees from the other 10 white pines distributed in China and adjacent areas (i.e., *P. wallichiana*, *P. kwangtungensis*, *P. fenzeliana*, *P. bhutanica*, *P. dabeshanensis*, *P. wangii*, *P. pumila*, *P. sibirica*, *P. morrisonicola*, and *P. koraiensis*) were also sampled for phylogeographic analysis. The detailed sampling information is shown in Table 1.

**Table 1:** Geographic locations and sample sizes of the Asian white pines sampled in this study.

Species	Subdivisions^1^	Population name/location	Sample	Latitude	Longitude
			size	(°N)	(°E)
*Pinus armandii*	QinLing-Daba Mts.(QDM)	DB/Diebu, Gansu	3	34.01	103.92
		ZQ/Zhouqu, Gansu	4	33.82	104.27
		WX/Wenxian, Gansu	19	32.98	104.56
		MX/Meixian, Shanxi	23	34.12	107.7
		LB/Liuba, Shanxi	5	33.63	106.73
		NS/Ningshan, Shanxi	6	33.45	108.47
		XS/Xingshan, Hubei	17	31.47	110.3
		SNJ/Shennongjia, Hubei	6	31.69	110.55
		HN/Lushi, Henan	5	33.92	110.7
		WY/Wanyuan, Sichuan	13	32.07	108.12
		GY/Guangyuan, Sichuan	15	32.56	106.16
		JY/Jiangyou, Sichuan	14	31.94	104.76
		BX/Baoxing, Sichuan	15	30.41	102.76
	Himalaya-HengDuan Mts.(HHM)	KD/Kangding, Sichuan	23	30.05	101.96
		CML/Muli, Sichuan	15	28.8	101.18
		CY/Chayu, Xizang(Tibet)	23	28.78	97.53
		ML/Milin, Xizang(Tibet)	15	29.44	94.81
		LZ/Linzhi, Xizang(Tibet)	17	29.94	94.75
		BM/Bomi, Xizang(Tibet)	14	29.92	95.46
		GS/Gongshan, Yunnan	15	27.78	98.55
		DQ/Deqin,Yunnan	15	28.31	98.97
	Yungui Plateau (YGP)	YH/Yuhu, Yunnan	21	26.12	99.97
		LS/Lushui, Yunnan	15	25.96	98.75
		TC/Tengchong, Yunnan	15	25.5	98.55
		CS/Cangshan, Yunnan	15	25.66	100.12
		MY/Miyi, Sichuan	15	27.05	102.01
		SM/Songming, Yunnan	16	25.26	103.04
		XY/Xingyi, Guizhou	20	25	104.79
*P. armandii* var. *masteriana*	Taiwan Island	DTS/Datashan, Taiwan	9	23.53	120.82
		YS/Yushan, Taiwan	7	23.47	120.94
*P. wallichiana*		JL/Jilong, Xizang(Tibet)	5	28.92	85.38
		YD/Yadong,Xizang(Tibet)	5	27.5	88.97
		PAK2/Naltar Bala, Pakistan	5	36.17	74.18
		PAK7/Rama Lake, Pakistan	5	35.33	74.79
*P. kwangtungensis*		LB/Libo, Guizhou	3	25.35	107.95
		YZ/Yizhang, Hunan	2	24.98	112.85
		WM/Wuming Mts, Guangxi	2	23.57	108.38
*P. fenzeliana*		HI/Hainan Island	2	18.88	109.66
		JFS/Nanchuan, Chongqing	6	29	107.09
*P. bhutanica*		LZ/Linzhi, Xizang(Tibet)	5	29.94	94.75
*P. dabeshanensis*		YX/Yuexi, Anhui	2	31.01	116.07
		JZ/Jinzhai, Anhui	1	31.4	115.6
		THC/Taohuachong, Hubei	1	30.97	116.06
*P. wangii*		MLP/Molipo, Yunnan	5	23.22	104.81
*P. pumila*		WTE/Changbai, Jilin	5	41.73	127.86
*P. sibirica*		KNS/Kanasi, Xinjiang	5	48.71	87.07
*P. morrisonicola*		NT/Nantou, Taiwan	1	23.91	120.68
		ML/Miaoli, Taiwan	1	24.53	120.94
*P. koraiensis*		CBS/Changbai Mts., Jilin	4	42.33	127.27

### DNA extraction, PCR amplification, and sequencing

Total genomic DNA was extracted from silica gel-dried leaves using the modified CTAB method [25] or DNAsecure Plant Kit (Tiangen). We initially screened 22 primer pairs for polymorphism by using a discovery panel composed of 20 individuals that represent all major geographic subdivisions of *P. armandii*. Two chloroplast fragments, *atp*H-*atp*I and *trn*C-*trn*D, and three mitochondrial fragments, *cox*1, *nad*5 intron 1, and *nad*7 intron1 were finally chosen for subsequent analysis in all sampled individuals. The five DNA segments were amplified with the primers used in previous studies [13], [26]–[29]. The PCR mixture and amplification program followed the protocols of Wei et al. [30], except that an annealing temperature of 50–55°C was used and the elongation time was adjusted according to the different markers. The PCR products were purified with PEG8000. Both the *cox*1 and *nad*5 intron 1 strands were sequenced using the amplification primers in all samples. The *atp*H-*atp*I gene and *nad*7 intron1 were sequenced by using a forward primer and *trn*C-*trn*D by using a reverse primer because all the informative polymorphisms could be covered by single primer sequencing. Sequencing was conducted with an ABI Prism 3730xl sequencer (PE Applied Biosystems). The sequences reported in this study were deposited in GenBank under accession numbers KF800948 - KF801058.

### Gene genealogy

Nucleotide sequences were aligned using CLUSTAL X [31] and manually adjusted in BioEdit v7.05.3 [32]. Since organellar genomes in plants generally do not recombine and are uniparentally inherited, we treated the two cpDNA markers and the three mtDNA markers as a single locus each. A polymorphic site caused by polyG (9–12 bp) in *nad*5 was excluded due to some low quality reads. All phylogenetically informative gaps (indels) were coded as single mutation events for analyses. Unique haplotypes were determined using the program DnaSP v5 [33]. All the rare haplotypes were verified by re-extracting DNA, re-amplifying and re-sequencing both strands.

NETWORK 4.611 (http://www.fluxus-engineering.com) was used for generating haplotype networks by the median-joining network algorithm. *P. koraiensis* was used as the outgroup because of its relatively distant relationship with the southern white pines (unpublished). Substitutions and indels were considered to evolve with equal possibility.

### Population genetic analyses

The spatial variance in haplotype distributions was analyzed using SAMOVA 1.0 (http://web.unife.it/progetti/genetica/Isabelle/samova.html) [34]. It implements a simulated annealing approach to define groups of populations (K) that are geographically homogenous and maximally differentiated from each other. The K values ranging from 2 to 9 were tested to search for the K that gave the highest *F*_CT_.

Nuclotide diversity (π) and Watterson's parameter (θ_W_) were estimated using DnaSP v5 [33]. The program PERMUTE [35] (http://www.pierroton.inra.fr/genetics/labo/software) was used for calculating the average gene diversity within populations (*H*_S_), total gene diversity (*H*_T_), and two measures of population differentiation (*G*_ST_, *N*_ST_). The two estimates of population divergence were compared using a permutation test with 1000 permutations. Genetic differentiation among populations and groups (identified by SAMOVA) was estimated by the analysis of molecular variance (AMOVA) with significance tests based on 10, 000 permutations using Arlequin 3.11 [36]. Population expansion was examined using the mismatch distribution analysis (MDA). For each model, goodness-of-fit, based on the sum of squared deviations (SSD), was tested using parametric demographic expansion bootstrapping with 1000 replicates. In addition, we also calculated Tajima's *D* [37] and Fu's *F*_S_ [38] to infer potential expansions. All the demographic tests were performed using Arlequin 3.11. When sudden expansions were detected, the MDA-derived expansion parameter (τ) was converted to absolute expansion time (*t*, in number of generations) according to the equation τ = 2*ut* [39], where *u* is the mutation rate per generation for the entire analyzed sequence. We calculated *u* according to *u* = μ*kg*, where μ is the substitution rate per nucleotide site per year, *k* is the average sequence length of the DNA region under study, and *g* is the generation time in years. We assumed that pines take 25–100 years to reach full seed production [40]. Substitution rate was obtained from the clock-calibrated BEAST (see the Results section).

### Phylogenetic analyses and estimates of divergence time

The evolutionary relationships of the chlorotypes and mitotypes were reconstructed with Maximum parsimony (MP), Maximum likelihood (ML), and Bayesian inference (BI) using PAUP*4.0b10 [41], PHYML2.4.4 [42] and MrBayes 3.1 [43], respectively. All phylogenetically informative gaps (indels) were coded as single mutation events. The final alignment was 1373 bp in length. The evolutionary models of GTR+I and GTR+I+G for cpDNA and mtDNA phylogenetic analyses, respectively, were determined by the Akaike information criterion (AIC) implemented in MrModeltest v2.3 [44]–[45]. All characters were treated as unordered and equally weighted in the MP analysis. A heuristic search strategy with 1000 replicates of random addition of sequences, tree-bisection-reconnection (TBR) branch-swapping and MULTREES options was involved in the MP and ML analyses. To assess the relative support for monophyletic groups, bootstrap analysis was conducted with 100 replicates using the same heuristic search settings as descripted above. In the Bayesian analysis, one cold and three incrementally heated Markov chain Monte Carlo (MCMC) chains were run simultaneously for 1,000,000 generations. Trees were sampled every 100 generations with the first 2500 samples discarded as burn-in. To establish the divergence time of the Asian white pines, a subset of our initial cpDNA data and five additional GenBank sequences from *P. monophylla* (NC_011158.4) and *P. maximartinezii* (JN854184.1) in Section *Parrya*, *P. bungeana* (JN854223.1) and *P. gerardiana* (NC_011154.4) in Subsection *Gerardianae*, and a North American white pine, *P. lambertiana* (FJ899577.1), were combined and reanalyzed with the aim of obtaining a useful calibration point. Phylogenetic trees were reconstructed with MP, ML, and BI by using the methods mentioned above. In this phylogenetic analysis, the two species from Section *Parrya* were used as outgroups due to the sister relationship between section *Quinquefolia* and section *Parrya* [23].

A Bayesian approach was employed to estimate the divergence time of each major group in the chlorotype phylogeny with BEAST v1.6.1 [46], using a relaxed uncorrelated lognormal clock model and the Yule model of speciation. Due to the lack of credible fossils from *P. armandii* or closely related species, we employed the crown node age of the two monophyletic sections *Quinquefolia* and *Parrya* (subgen. *Strobus*) reported by Willyard et al. [17]. We used a normal distribution centered at 35 Ma with a standard deviation of 1.0 Ma. This gave a central 95% range of 33–37 Ma, which is within the ranges reported by the two studies [17], [47] from independent fossil calibrations. The MCMC analysis was run for 30,000,000 generations. We sampled 1000 trees with the first 50% treated as burn-in. The output file was analyzed using TRACER 1.5 (http://beast.bio.ed.ac.uk/Tracer). The measures of effective sample sizes (ESS) were used to determine the Bayesian statistical significance of each parameter.

## Results

### Chlorotype distribution and genealogy

The lengths of the two cpDNA regions (*atp*H-*atp*I and *trn*C-*trn*D) in the white pines were found to be 674–686 bp and 893–954 bp, respectively. The combined cpDNA dataset is 1565 bp in length. Six indels and 13 substitutions were detected and resulted in 20 chlorotypes (C1–C20) among the trees surveyed. Of those, C1, C6 and C9 were the most common chlorotypes. *P. armandii* harbored 13 chlorotypes C1–C13. C14 is unique to the two Taiwanese white pines, *P. armandii* var. *masteriana* and *P. morrisonicola*, as well as the two north Asian species *P. pumila* and *P. sibirica*. C15 and C16 were fixed in *P. wallichiana*. C17 and C18 are rare chlorotypes, unique to JFS of *P. fenzeliana*. *P. koraiensis* harbored C19 and C20.

**Figure 2:**
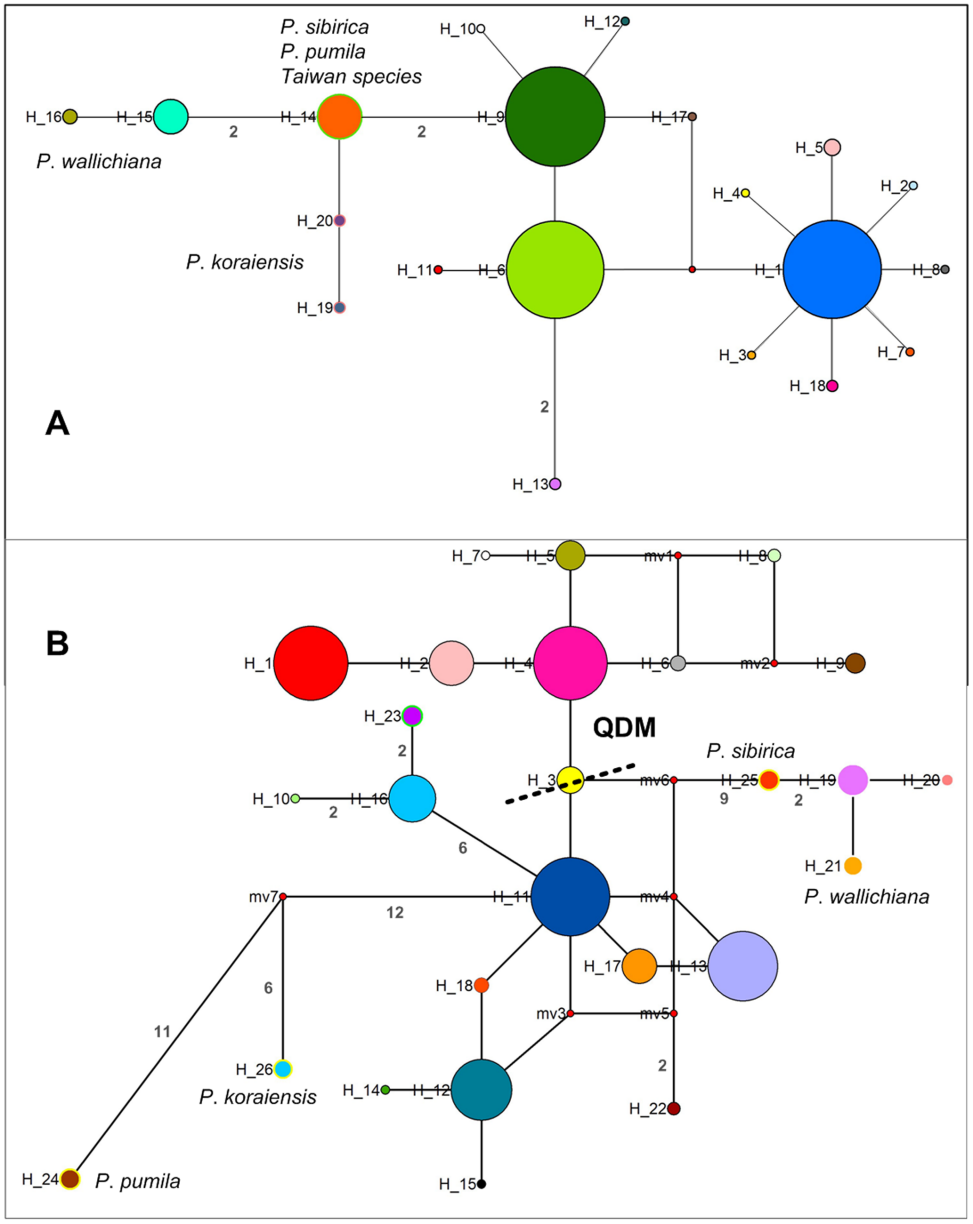
NETWORK-derived genealogical relationship of chlorotypes (A) and mitotypes (B). The sizes of the circles in the network are proportional to the observed frequencies of the haplotypes. The number of mutational steps larger than one is shown next to the solid line between haplotypes.

A strong geographic pattern of chlorotype variation was observed in *P. armandii*. SAMOVA showed that the value of *F*_CT_ reached a plateau at 0.87 for four groups (K = 4). The two populations (DTS and YS) of *P. armandii* var. *masteriana* from Taiwan were separated as a distinct group. The other three groups from mainland China also had a strong geographic basis, generally corresponding to the Qinling-Daba Mountains (DB, ZQ, WX, MX, LB, NS, XS, SNJ, HN, WY, GY, JY, and BX), Himalaya-Hengduan Mountains (KD, CML, DQ, CY, ML, LZ, BM, and GS) and Yungui Plateau (YH, LS, TC, CS, MY, SM, and XY). Hereafter, we designate these three groups as QDM, HHM, and YGP, respectively. Each group harbored a dominant chlorotype (Fig. 1A). C1 was unique to QDM. C6 and C9 dominated in HHM and YGP, respectively. In addition, C6 occurred at a low frequency in several QDM populations (BX, JY, WY, and XS) and *P. bhutanica*, *P. dabeshanensis*, *P. fenzeliana*, *and P. wangii*. C9 only fixed in southern populations, including the three subtropical white pines, *P. kwangtungensis*, *P. wangii*, and *P. fenzeliana*. It is notable that all the three common chlorotypes were present in the KD population with high frequency (Fig. 1A). The chlorotype network delineated three major clades of *P. armandii* populations in mainland China (Fig. 2A), which broadly correspond to the three geographic subdivisions (QDM, HHM, and YGP).

### Mitotype distribution and genealogy

The sizes of the three mtDNA markers, *cox*1, *nad*5 intron 1, and *nad*7 intron 1, are 1225 bp, 1035–1042 bp and 809–1244 bp, respectively. Five individuals from populations WX, WY, and BX of *P. armandii* were excluded due to a polymorphic nucleotide site in the *nad*5 intron 1 and 15 individuals from *P. armandii* var. *masteriana* because of failed amplification of the *nad*7 intron 1. A total of 24 indels and 28 substitutions were identified for the concatenated mitochondrial DNA sequence which yielded 26 mitotypes (M1–M26). Of those, *P. armandii* harbored 17 (M1–M17), and four of them (M4, M11, M12, and M16) are shared across species. M18 was fixed in the two Taiwanese white pines (*P. armandii* var. *masteriana* and *P. morrisonicola*). M19–M26 were restricted to the remaining white pines. No more than three mitotypes were found in a single population, with the exception of XS, which harbored five (M4–M8).

QDM possessed ten mitotypes (M1–M10), and eight (M1–M2, M5–M10) were unique. Of these, M1 occurred in all populations except three in the east. M3 was only found in nine individuals from three populations (WX, HN, and KD) across a large geographic range. M4 dominated in eastern YGP, but it was also present in eastern QDM (MX, XS, NS) and in the JFS population of *P. fenzeliana*. M12 and M13 predominated in HHM. M16 occurred at high frequency in eastern YGP and shared with its adjacent population (LB) from *P. kwangtungensis*. M11 had a disjunct distribution between the southeastern margin of the QTP (*P. armandii*) and Dabie Mountains in eastern China (*P. dabeshanensis*).

Unlike the chlorotypes, the mitotype network did not show clear geographic relationships among QDM, HHM and YGP (Fig. 2B). It is compatible with the SAMOVA of mtDNA, in which no plateau value of *F*_CT_ was obtained. Nevertheless, two clusters linked by M3 were broadly delineated in *P. armandii*, one comprised of all but one (M10) mitotype from the QDM lineage and the other included all the mitotypes from the HHM and YGP lineages except M4, which nested in the QDM cluster. In the latter cluster, M11 (KD, CML, YH, CS, and TC) was placed in the interior position, directly linked by M3 (KD and HN), M16 (CS, MY, SM, XY, and LB), M17 (DQ), and M18 (Taiwan) (Fig. 2B). In theory, haplotypes with an interior position in a network are often older than haplotypes on the tips [48]. In addition, the close relationship of M11 with *P. koraiensis* (M26), which was used as an outgroup in the network analysis, also suggests that it is old.

### Genetic diversity and population differentiation of P. armandii

Both cpDNA and mtDNA showed high levels of total genetic diversity (*H*_T_) over populations of *P. armandii* from mainland China (0.692 for cpDNA, 0.886 for mtDNA), but the levels of average within-population diversity (*H*_S_) were much lower (0.157 and 0.239 for cpDNA and mtDNA, respectively; Table 2). Population differentiation was remarkably high, with *G*_ST_ values being 0.773 and 0.730 for cpDNA and mtDNA, respectively (Table 2). The permutation tests of cpDNA showed that *N*_ST_ was significantly higher than *G*_ST_ (*P*<0.01), suggesting a strong phylogeographic structure in *P. armandii*. However, *N*_ST_ was not significantly greater than *G*_ST_ at mtDNA loci. AMOVA showed that 83.14% and 82.31% of the total cpDNA and mtDNA variation respectively resided among populations (Table 3).

**Table 2:** Total genetic diversity, average genetic diversity within populations and population differentiation estimates in *Pinus armandii*.

Region	cpDNA				mtDNA			
	*H*_T_	*H*_S_	*N*_ST_	*G*_ST_	*H*_T_	*H*_S_	*N*_ST_	*G*_ST_
QDM	0.171	0.163	0.053	0.048	0.689	0.290	0.737*	0.579
HHM	0.334	0.241	0.231	0.278	0.833	0.061	0.955	0.927
YGP	0.055	0.051	0.074	0.068	0.788	0.349	0.430	0.557
Mainland China	0.692	0.157	0.860**	0.773	0.886	0.239	0.790	0.730
Entire range	0.730	0.147	0.889**	0.799	nc	nc	nc	nc

** extremely significant (Nst>Gst, P<0.01);

* significant (Nst>Gst, P<0.05); nc, not be calculated due to single individual in YS population.

**Table 3:** Analysis of molecular variance (AMOVA) of *Pinus armandii* based on cpDNA and mtDNA haplotypes.

Source of variation	d.f.		Sum of squares		Variance		Percentage		Fixation indices	
					components		of variation			
	cpDNA	mtDNA	cpDNA	mtDNA	cpDNA	mtDNA	cpDNA	mtDNA	cpDNA	mtDNA
Mainland China										
Among populations	27	27	242.95	410.83	0.63	1.07	83.14	82.31		
Within populations	371	366	47.10	84.30	0.13	0.23	16.86	17.69	*F*_ST_ = 0.83	*F*_ST_ = 0.82
Total	398	393	290.04	495.13	0.75	1.30				
Divided into three regions										
(QDM, HHM, and YGP)										
Among regions	2	2	233.13	195.78	0.88	0.67	85.73	44.45	*F*_SC_ = 0.13	*F*_SC_ = 0.72
Among populations within	25	25	9.81	215.05	0.02	0.61	1.86	40.27	*F*_ST_ = 0.88	*F*_ST_ = 0.85
regions									*F*_CT_ = 0.86	*F*_CT_ = 0.44
Within populations	371	366	47.10	84.30	0.13	0.23	12.42	15.28		
Total	398	393	290.04	495.13	1.02	1.51				
Divided into two regions										
(North and South)										
Among regions	1	1	189.55	159.46	1.01	0.82	79.38	47.60	*F*_SC_ = 0.68	*F*_SC_ = 0.75
Among populations within	26	26	53.39	251.37	0.14	0.68	10.69	39.10	*F*_ST_ = 0.82	*F*_ST_ = 0.87
regions									*F*_CT_ = 0.44	*F*_CT_ = 0.48
Within populations	371	366	47.10	84.30	0.13	0.23	9.93	13.30		
Total	398	393	290.04	495.13	1.28	1.73				

At the regional level, contrasting patterns of genetic diversity and population differentiation were observed at cpDNA and mtDNA loci (Table 2, 3). For cpDNA, the level of total genetic diversity (*H*_T_ = 0.055–0.334) and of population differentiation (*G*_ST_ = 0.048–0.278) were relatively low in each of the three subdivisions (QDM, HHM, and YGP). Accordingly, the hierarchical analysis of molecular variance (AMOVA) showed a high level of variation among regions (85.73%) and extremely low level of variation (1.86%) among populations within regions. Taiwan populations were monomorphic. In contrast, the total genetic diversity (*H*_T_ = 0.689–0.833) and population differentiation (*G*_ST_ = 0.557–0.927) were very high in mtDNA. The partitioning of the mtDNA variation among regions and among populations within regions were 44.45% and 40.27%, respectively. Among the three geographic subdivisions, HHM had the highest level of genetic diversity and population differentiation at both cpDNA and mtDNA (Table 2).

### Phylogenetic analyses and divergence time estimate

**Figure 3:**
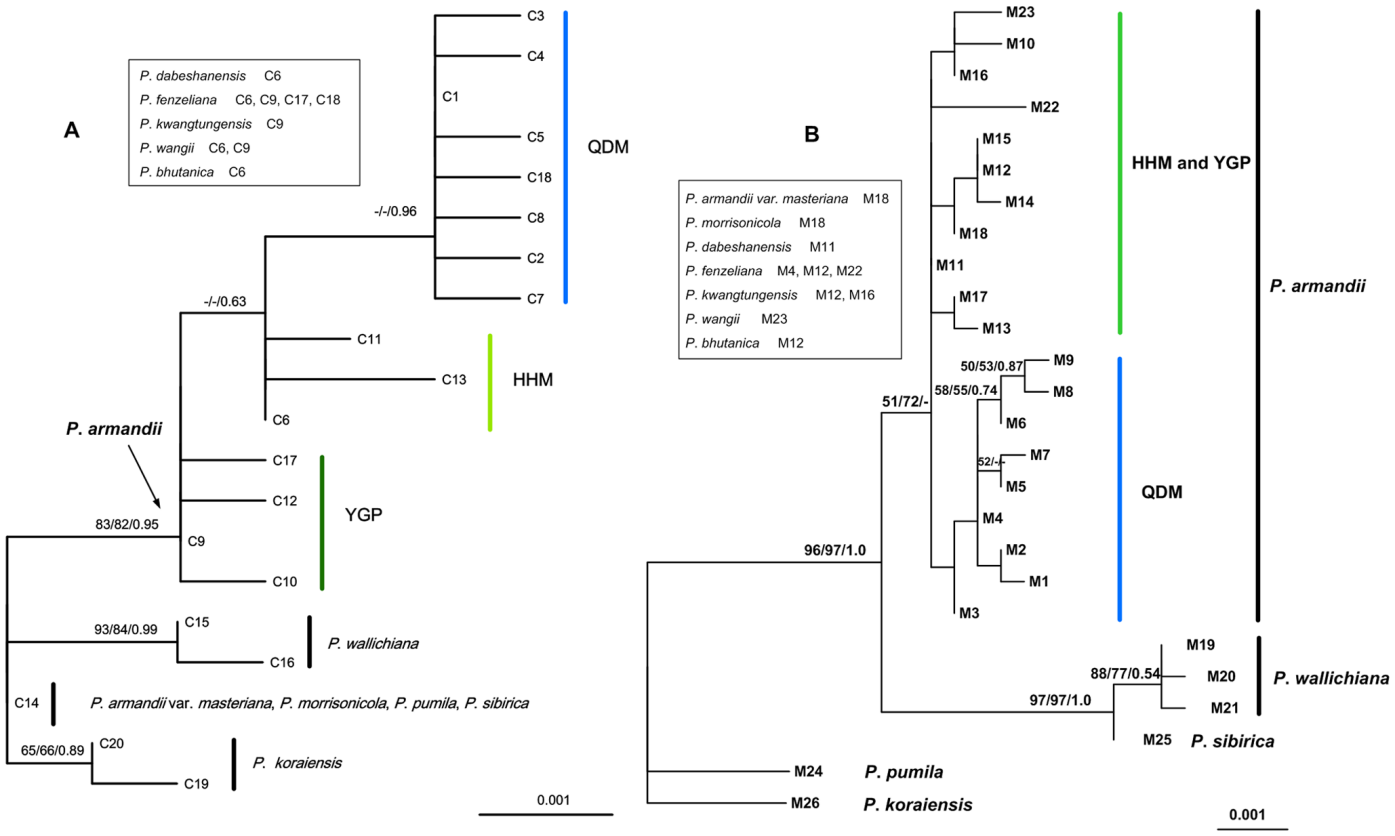
Phylogenetic topography obtained by analysis of chlorotypes (A) and mitotypes (B). Numbers above the branches are bootstrap values greater than 50% for ML (left) and MP (middle) analyses, and Bayesian posterior probabilities (right). A dash indicates a bootstrap value lower than 50%. The other species included in the *Pinus armandii* clade are shown in the box.

Both cpDNA and mtDNA phylogenies (Fig. 3A, B) indicated that the Himalayan pine, *P. wallichiana*, was distantly related to the southern pines in mainland China, i.e., *P. armandii*, *P. bhutanica*, *P. dabeshanensis*, *P. fenzeliana*, *P. kwangtungensis*, and *P. wangii*. In the cpDNA tree, three lineages corresponding to QDM, HHM, and YGP, were resolved within the clade including *P. armandii*. The two Taiwanese white pines clustered together with two North Asian pines, *P. sibirica* and *P. pumila*, and showed a sister relationship to *P. armandii*. In the mtDNA tree, however, the two white pines in Taiwan were included in the *P. armandii* clade.

**Figure 4:**
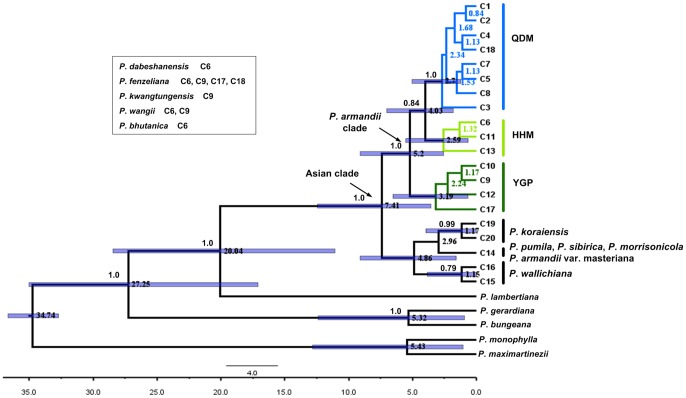
Phylogenetic chronogram of the chlorotypes generated from BEAST. Numbers above the branches indicate the Bayesian posterior probabilities greater than 0.5. Median ages of nodes are shown, with horizontal bars indicating the 95% highest posterior density intervals. The other species included in the *Pinus armandii* clade are shown in the box.

Molecular dating showed that the age of the split between the Asian and North American white pines was about 20.04 Ma (95% highest posterior density (HPD) interval, 28.46 Ma – 11.09 Ma). The Asian white pines were divided into two lineages around 7.41 Ma (95% HPD interval, 12.44 Ma – 3.57 Ma). One lineage was composed of the widespread Himalayan pine *P. wallichiana*, three northern pines, *P. koraiensis*, *P. sibirica*, *P. pumila*, and two from Taiwan, *P. morrisonicola* and *P. armandii* var. *masteriana*, while the other was comprised of *P. armandii* and the other white pines in southern China. Within the *P. armandii* clade, YGP diverged at about 5.2 Ma (95% HPD interval, 9.09 Ma – 2.58 Ma). QDM and HHM were separated 4.03 Ma (95% HPD interval, 7.01 Ma – 1.84 Ma). All these estimates are shown in Fig. 4. The mean nucleotide substitution rate for the combined cpDNA region was estimated to be 0.47×10^−9^ per site per year.

### Demographic analyses

Under a model of sudden population expansion (*P*>0.05), for cpDNA, each of the three regions, QDM, HHM and YGP, showed a strongly unimodal mismatch distribution and nonsignificant SSD index values (Fig. S1), indicating a historical population expansion. However, Fu's *F*s and Tajima *D* tests suggest only QDM had significantly negative Fu's *F*_S_ and Tajima *D* values (*F*_S_ = −8.23581, *P*<0.001; *D* = −1.88930, *P*<0.05) (Table S1). Therefore, we can say with confidence that QDM experienced expansion. The expansion of QDM was estimated at 93 ka (a generation time of 25 y applied) and 23.2 ka (a generation time of 100 y applied). For mtDNA, none of them showed strongly unimodal mismatch distribution and significantly negative values of Tajima *D* or Fu's *F*_S_.

## Discussion

### Strong genetic differentiation of *P. armandii* and uplift of the QTP

Compared with the average genetic differentiation of conifers (cpDNA = 0.165; mtDNA = 0.764) [49] and those reported recently for other Pinaceae species (e.g., [50]–[55]), *P. armandii* exhibits high levels of total genetic diversity and significant population differentiation at both cpDNA (*H*_T_ = 0.692, *F*_ST_ = 0.83, *G*_ST_ = 0.773) and mtDNA loci (*H*_T_ = 0.886, *F*_ST_ = 0.82, *G*_ST_ = 0.730) (Table 2, 3). As a conifer with the capacity for long-distance pollen dispersal, the high level of the total cpDNA variation among regions (85.73%), and extremely low level among populations within regions (1.86%; Table 3), suggested that geography might play an important role in driving the genetic structure of *P. armandii* population.

The chlorotypes revealed three lineages of *P. armandii* in mainland China that geographically corresponding to the Qinling-Daba Mountains (QDM), Himalaya-Hengduan Mountains (HHM), and Yungui Plateau (YGP). Their break zone was observed along the southeastern margin of the QTP. The QDM lineage was separated from the two south lineages (HHM and YGP) at Kangding (KD) in western Sichuan, which is consistent with a previous provenance study [56]. Numerous high mountains and plateaus over 6,000 m above the sea level (a.s.l.), including the highest Gongga Mountain (7,556 m a.s.l.) (Fig. 1B), are located nearby and divide the Hengduan Mountains into northern and southern floristic sub-regions [57]. The genetic isolation of the HHM and YGP populations was consistent with the geographic transition of the Hengduan Mountains and the Yungui Plateau, two large natural geographic regions characterized by a series of parallel massive mountains and deeply carved valleys running roughly north to south and mid-elevation mountains oriented from west to east, respectively. It is likely that these massive mountains at the southeastern margin of the QTP imposed significant geographic barriers to the genetic exchange among different lineages of *P. armandii*. Active mountain building, induced by the recent uplift of the southeastern margin of the QTP, might strongly affect the genetic structure of *P. armandii*.

A close link was detected between the recent rapid uplift of the QTP and the differentiation of *P. armandii*. Based on our analysis of molecular dating, *Pinus armandii* split from the Himalayan pine *P. wallichiana* around 7.41 Ma (95% HPD interval, 12.44 Ma – 3.57 Ma). The subsequent divergence of *P. armandii* was dated to early Pliocene (5.2–4.03 Ma; Fig. 4). Although we need to be cautious with these estimates due to the limited data, they are concordant with a Miocene origin (10–20 Ma) of the two subsections *Strobus* and *Cembroides* that harbor the greatest number of soft pines [58]. Several lines of evidence suggest that the rapid uplift of the eastern part of the QTP took place around 13 to 5 Ma [59]–[60]. Dramatic geological changes induced by the QTP uplift contributed to the extremely complicated landscape in the eastern fringe, which likely acted as a major driving force for the separation of the three lineages of *P. armandii*. Divergent selection between populations in contrasting environments would accelerate their differentiation, and eventually promote allopatric speciation.

### Recent diversification of the subtropical white pines and Taiwan species

In wind-pollinated conifers with contrasting modes of inheritance, weaker population differentiation is often observed in pollen-mediated cpDNA than seed-mediated mtDNA (e.g., [30], [49]–[50], [61]). In *P. armandii*, such asymmetric differentiation between cpDNA and mtDNA was indeed observed at the regional scale. However, across its entire range, the cpDNA differentiation was surprisingly much more highly structured geographically than the mtDNA. The hierarchical AMOVA also showed that 85.73% and 44.45% of the cpDNA and mtDNA variation respectively resided among regions (Table 3), indicating more restricted pollen-mediated gene flow than seed-mediated gene flow among the three lineages. This unique pattern detected in *P. armandii* can likely be attributed to its evolutionary history and considerable gene flow via seed migration in the past.

Several lines of evidence, including the chlorotype composition of the KD population, a large number of common mitotypes and the dominant presence of the putative ancestral mitotype (M11), indicate that *P. armandii* originated in southwest China and probably in the eastern part of the QTP (Fig. 1). It is surprising that M11 and C6, which dominated in the southeastern margin of the QTP, were also found in eastern China (*P. dabeshanensis*). A similar disjunct distribution pattern was also observed in M12 and C6, between the HHM lineage of *P. armandii* and some populations of *P. fenzeliana* (HI), *P. kwantungensis* (YZ), and *P. wangii* (MLP) in southern China. It is likely that a large continuous population of white pines once existed from southwest to subtropical China, but now only persists in isolated geographic aggregates. Range disjunctions for plant species are often a consequence of Quaternary climate changes [2]. A scenario could be inferred that in tracking repeated Pleistocene glaciations, these montane white pines may have migrated up and down elevation gradients. During these necessary movements into glacial refugia, they experienced severe fragmentation and isolation. In addition, population bottlenecks might have occurred in *P. dabeshanensis* and led to its extremely small population size and low genetic diversity. This scenario was supported by the phylogeographic studies of *P. kwantungensis* [54]–[55], as well as their current distributions and morphological similarities. These white pines are scattered in the broadleaved evergreen forest region in subtropical China, a vast region where well-known centers of plant diversity and endemism exist [7], and can also often be found on isolated mountaintops, cliffs, or slopes. Some areas, such as the Dabie Mountains, Jinfo Mountains, Hainan Island, Nanling Mountains, and eastern Yungui Plateau are often postulated as glacial refugia for warm-temperate evergreen forest species [54]–[55], [62]–[64]. Long-term persistence in isolated refugia would promote their divergence and eventual speciation.

The origin of the florist affinity between Taiwan and southwestern China/Himalayas is a fascinating question regarding the biogeography of eastern Asia [30], [65]. *P. armandii* var. *masteriana* and *P. morrisonicola* are endemic to Taiwan. Their primary difference is that *P. armandii* var. *masteriana* (like *P. armandii* in mainland China) has wingless seeds whereas *P. morrisonicola* has winged seeds. In addition, *P. armandii* var. *masteriana* generally occurs at higher altitudes (1800–3300 m) than *P. morrisonicola* (300–2300 m) [18]. It is interesting that the two species share a single mitotype (M18) which is closely related to M11, suggesting they evolved from a common ancestor in mainland China. The ancestor was probably in eastern China due to the presence of M11 in the Dabie Mountains and the short distance from Taiwan. The Taiwanese populations were genetically less diverse in both cpDNA and mtDNA markers, which might correspond to a rather recent colonization. Unexpectedly, the two Taiwanese white pines shared the single chlorotype C14 with two North Asian white pines, *P. pumila* and *P. sibirica*, indicating a North Asian origin of their chloroplasts. The inconsistency between the cpDNA and mtDNA markers was observed in the cytoplasmic phylogenies (Fig. 3). Introgression via long-distance pollen dispersal may be the most probable cause of the incongruence. Furthermore, a single chloroplast introgression event might occur in the two white pines followed by a loss of genetic diversity through drift effects in an island population. One may argue that the long-distance dispersal is inconsistent with the idea of limited pollen dispersal via geographic barriers among the three lineages of *P. armandii* in southwest China. However, the complicated landscapes in southwest China, particularly numerous massive mountains, likely imposed more significant barriers to pollen dispersal than the Pacific islands for wind-pollinated trees. The current distribution of *P. pumila* in Japan provides further support for this supposition. Frequent introgression and hybridization is well documented in pine species [66]. Our results suggested that interspecific introgression might also occur between the eastern YGP populations of *P. armandii* and LB population of *P. kwantungensis*, as well as between the HHM populations of *P. armandii* and *P. bhutanica*. Our study demonstrated the importance of including more closely related species in phylogeographic research and the value of a clear evolutionary background for resolving the taxonomic uncertainties in the problematic white pines.

### Heterogeneous contributions of geography and climate changes to the genetic differentiation of Chinese white pines

Quaternary climate changes produced great changes in species distribution and population structure [2]. Nevertheless, the diversification patterns may be spatially varied. In the case of Chinese white pines, different genetic patterns were observed among populations from the Qinling-Daba Mountains, Himalaya-Hengduan Mountains, Yungui Plateau and southern China, which are diverse in geographic, climatologic and topographic features. The north facing Qinling Mountains oriented from east to west in central China form a natural dividing line between Northern and subtropical (Central/East/South) China and serve as a major geographic barrier to the cold, dry air coming in from the north. Thus, this region might be subjected to relatively stronger influences of Pleistocene glaciations. As expected, mismatch analysis and the indices of Fu's F* and Tajima *D* suggested that only the QDM lineage of *P. armandii* has undergone a sudden population expansion which was estimated to have occurred around 23–93 ka, close to the last glacial maximum (18–24 ka BP). The large number of private haplotypes and high levels of nucleotide diversity of the SNJ and XS populations (Table S1, S2, S3) suggest that the eastern Daba Mountains likely served as a main refugium for the QDM lineage. Among the three lineages of *P. armandii*, the HHM lineage exhibits the highest level of genetic diversity and population differentiation (Table 2). This could be attributed to the extremely dissected topography of the HHM, which acted as significant barriers to gene flow.

It is noteworthy that disjunct mitotypes (e.g., M11, M12) found in the eastern margin of the QTP and the subtropical China are missing in the eastern Yungui Plateau. Instead, the eastern YGP populations (MY, SM, and XY) of *P. armandii* are dominated by M4 and M16. It is likely that the relatively less complex geomorphological configuration of the YGP not only contributed to the low genetic diversity and population differentiation in that region (Table 2), but also promoted the expansion of some putative adaptive alleles. In contrast, subtropical China is characterized by extremely rugged low mountains and relatively stable environments, which may have favored the preservation of relict lineages. Phylogeographic studies of *P. kwantungensis* have revealed many glacial refugia in this region and high levels of population differentiation at both cpDNA (*G*st = 0.63 vs. 0.068 in YGP) and mtDNA loci (*G*st = 0.75 vs. 0.557 in YGP) [54]–[55]. These findings highlighted the significance of both Quaternary climate changes and geographic factors in shaping the current genetic structures of Chinese white pines.

## Supporting Information

Figure S1 **Mismatch distributions for the different subdivisions of** ***Pinus armandii*** **.** Capital and lower case letters indicated the distributions of chlorotypes and mitotypes, and the black and gray lines represent the expected and observed mismatch distributions, repectively. Aa: mainland China; Bb: QDM; Cc: YGP; Dd: HDM.
(TIF)

Table S1 Tajima's *D* and Fu's *F_S_* neutrality tests and nucleotide diversity of each population and geographic subdivision of *Pinus armandii* at cpDNA and mt DNA loci.
(DOC)

Table S2 Chlorotype distribution in each population of *Pinus armandii* and the other white pines in this study.
(DOC)

Table S3 Mitotype distribution in each population of *Pinus armandii* and the other white pines in this study.
(DOC)
