# Supplementary figures and images for: Phylogeography of Pinus armandii and Its Relatives: Heterogeneous Contributions of Geography and Climate Changes to the Genetic Differentiation and Diversification of Chinese White Pines
Source: PLoS One. 2014 Jan 21;9(1):e85920. doi: 10.1371/journal.pone.0085920 (PMC3897548; doi:10.1371/journal.pone.0085920)

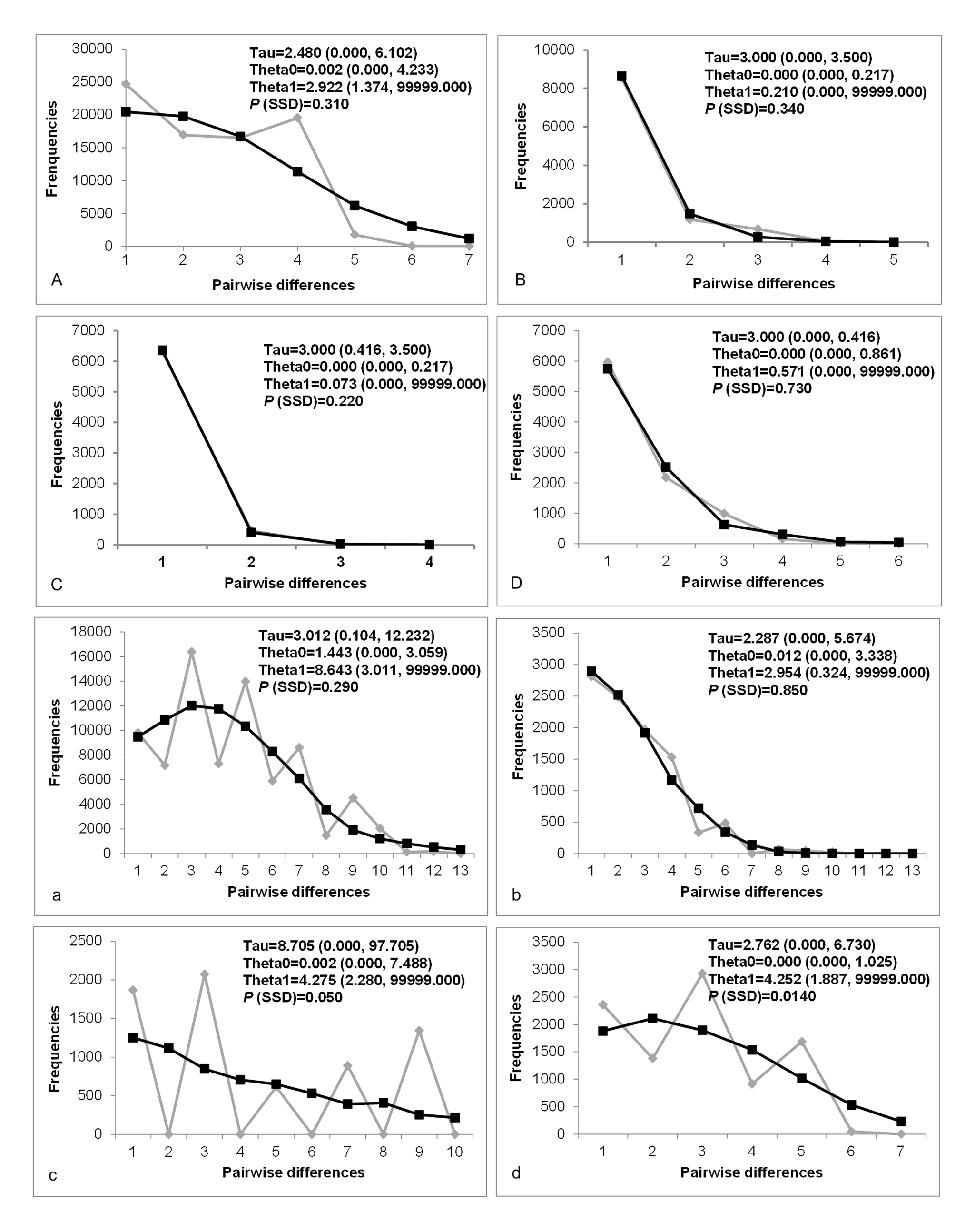

Supplement: Figure S1 — Mismatch distributions for the different subdivisions of Pinus armandii . Capital and lower case letters indicated the distributions of chlorotypes and mitotypes, and the black and gray lines represent the expected and observed mismatch distributions, repectively. Aa: mainland China; Bb: QDM; Cc: YGP; Dd: HDM. (TIF) [file pone.0085920.s001.tif]
