# Supplementary material for: Phylogeography of Pinus armandii and Its Relatives: Heterogeneous Contributions of Geography and Climate Changes to the Genetic Differentiation and Diversification of Chinese White Pines
Source: PLoS One. 2014 Jan 21;9(1):e85920. doi: 10.1371/journal.pone.0085920 (PMC3897548; doi:10.1371/journal.pone.0085920)
Supplement: Table S1 — Tajima's D and Fu's FS neutrality tests and nucleotide diversity of each population and geographic subdivision of Pinus armandii at cpDNA and mt DNA loci. (DOC) [file pone.0085920.s002.doc]

| Population  (Subdivision) | **cpDNA** | | | |  | **mtDNA** | | | |
| --- | --- | --- | --- | --- | --- | --- | --- | --- | --- |
|  | *D* | *F_S_* | π | *θ*_W_ |  | *D* | *F_S_* | π | *θ*_W_ |
| DB | 0.00000 | nc | 0.0000 | 0.0000 |  | 0.00000 | nc | 0.00000 | 0.0000 |
| ZQ | 0.00000 | nc | 0.0000 | 0.0000 |  | 0.00000 | nc | 0.00000 | 0.0000 |
| WX | -1.16480 | -0.83782 | 0.00007 | 0.00018 |  | -1.09557 | -0.17613 | 0.00018 | 0.00029 |
| MX | -1.51496* | -2.02722* | 0.00011 | 0.00035 |  | 0.40524 | 0.37245 | 0.00021 | 0.00018 |
| LB | 0.00000 | nc | 0.0000 | 0.0000 |  | -0.81650 | 0.09021 | 0.00013 | 0.00016 |
| NS | 0.85057 | 0.62543 | 0.00034 | 0.00028 |  | -1.13197 | 0.95213 | 0.00022 | 0.00029 |
| XS | -1.57683 | -1.42016 | 0.00037 | 0.00076 |  | -0.39771 | -1.26365 | 0.00034 | 0.00039 |
| SNJ | 0.93302 | -0.00275 | 0.00021 | 0.00028 |  | -1.13197 | 0.95213 | 0.00022 | 0.00029 |
| HN | 0.00000 | nc | 0.0000 | 0.0000 |  | 0.00000 | nc | 0.00000 | 0.00000 |
| WY | -0.35040 | 1.31652 | 0.00036 | 0.00041 |  | 0.67135 | 0.77852 | 0.00014 | 0.00011 |
| GY | 0.00000 | nc | 0.0000 | 0.0000 |  | 0.74210 | 0.90859 | 0.00014 | 0.00010 |
| JY | -1.48074 | 0.29648 | 0.00018 | 0.00040 |  | 0.32440 | 0.64281 | 0.00012 | 0.00010 |
| BX | -1.49051 | 0.23493 | 0.00017 | 0.00039 |  | -1.48074 | 0.29648 | 0.00009 | 0.00021 |
| **QDM** | **-1.88930^**^** | **-8.23581^***^** | **0.00016** | **0.00092** |  | **-0.13530** | **-1.40955** | **0.00036** | **0.00054** |
| KD | 0.54359 | 0.77752 | 0.00083 | 0.00069 |  | -0.21321 | 0.26588 | 0.00008 | 0.00009 |
| CML | 1.50272 | 1.31784 | 0.00034 | 0.00020 |  | 0.00000 | nc | 0.00000 | 0.00000 |
| CY | -0.21313 | -0.15289 | 0.00031 | 0.00035 |  | 0.00000 | nc | 0.00000 | 0.00000 |
| ML | 0.00000 | nc | 0.00000 | 0.00000 |  | 0.00000 | nc | 0.00000 | 0.00000 |
| LZ | 0.00000 | nc | 0.00000 | 0.00000 |  | -1.70573* | 0.70838 | 0.00012 | 0.00029 |
| BM | 0.00000 | nc | 0.00000 | 0.00000 |  | 0.00000 | nc | 0.00000 | 0.00000 |
| GS | 0.00000 | nc | 0.00000 | 0.00000 |  | 0.00000 | nc | 0.00000 | 0.00000 |
| DQ | -0.51271 | 1.18757 | 0.00032 | 0.00039 |  | 0.00000 | nc | 0.00000 | 0.00000 |
| **HHM** | **-1.34653** | **-2.25743** | **0.00032** | **0.00081** |  | **0.61673** | **1.99282** | **0.00059** | **0.00030** |
| YH | -0.13252 | 0.34111 | 0.00016 | 0.00018 |  | 1.95847 | 3.07469 | 0.00034 | 0.00018 |
| LS | 0.00000 | nc | 0.00000 | 0.00000 |  | 0.00000 | nc | 0.00000 | 0.00000 |
| TC | 0.00000 | nc | 0.00000 | 0.00000 |  | 0.00000 | nc | 0.00000 | 0.00000 |
| CS | 0.00000 | nc | 0.00000 | 0.00000 |  | 0.15732 | 0.86498 | 0.00032 | 0.00030 |
| MY | 0.00000 | nc | 0.00000 | 0.00000 |  | 1.63118 | 3.62666 | 0.00047 | 0.00030 |
| SM | 0.00000 | nc | 0.00000 | 0.00000 |  | 1.50995 | 3.60049 | 0.00045 | 0.00030 |
| XY | -1.16439 | -0.87930 | 0.00006 | 0.00018 |  | 1.86741 | 4.06151 | 0.00047 | 0.00028 |
| **YGP** | **-1.22442** | **-2.99287^**^** | **0.00004** | **0.00024** |  | **1.59565** | **3.74896** | **0.00056** | **0.00031** |
| DTS | 0.00000 | nc | 0.00000 | 0.00000 |  | nc | nc | nc | nc |
| YS | 0.00000 | nc | 0.00000 | 0.00000 |  | nc | nc | nc | nc |
| **Taiwan** | **0.00000** | **nc** | **0.00000** | **0.00000** |  | nc | nc | nc | nc |

Table S1 Tajima’s *D* and Fu’s *F_S_* neutrality tests and nucleotide diversity of each population and geographic subdivision of *Pinus* *armandii* at cpDNA and mt DNA loci.

Note: * 0.01<p<0.05; ** 0.001<p<0.01; *** p<0.001; nc, can not be calculated if only one allele or single individual in a population; π, nucleotide diversity (Nei & Li, 1979); θ_W_, Watterson’s parameter (Watterson, 1975)
