# Supplementary material for: Phylogeography of Pinus armandii and Its Relatives: Heterogeneous Contributions of Geography and Climate Changes to the Genetic Differentiation and Diversification of Chinese White Pines
Source: PLoS One. 2014 Jan 21;9(1):e85920. doi: 10.1371/journal.pone.0085920 (PMC3897548; doi:10.1371/journal.pone.0085920)
Supplement: Table S2 — Chlorotype distribution in each population of Pinus armandii and the other white pines in this study. (DOC) [file pone.0085920.s003.doc]

Table S2 Chlorotype distribution in each population of *P*. *armandii* and the other white pines in this study.

| Species | Pop |  | Chlorotype | | | | | | | | | | | | | | | | | | | |
| --- | --- | --- | --- | --- | --- | --- | --- | --- | --- | --- | --- | --- | --- | --- | --- | --- | --- | --- | --- | --- | --- | --- |
|  |  | n | C1 | C2 | C3 | C4 | C5 | C6 | C7 | C8 | C9 | C10 | C11 | C12 | C13 | C14 | C15 | C16 | C17 | C18 | C19 | C20 |
| *P. armandii* | DB | 3 | 3 |  |  |  |  |  |  |  |  |  |  |  |  |  |  |  |  |  |  |  |
|  | ZQ | 4 | 4 |  |  |  |  |  |  |  |  |  |  |  |  |  |  |  |  |  |  |  |
|  | WX | 19 | 18 | 1 |  |  |  |  |  |  |  |  |  |  |  |  |  |  |  |  |  |  |
|  | MX | 23 | 21 |  | 1 | 1 |  |  |  |  |  |  |  |  |  |  |  |  |  |  |  |  |
|  | LB | 5 | 5 |  |  |  |  |  |  |  |  |  |  |  |  |  |  |  |  |  |  |  |
|  | NS | 6 | 4 |  |  |  | 2 |  |  |  |  |  |  |  |  |  |  |  |  |  |  |  |
|  | XS | 17 | 13 |  |  |  | 2 | 1 | 1 |  |  |  |  |  |  |  |  |  |  |  |  |  |
|  | SNJ | 6 | 5 |  |  |  |  |  |  | 1 |  |  |  |  |  |  |  |  |  |  |  |  |
|  | HN | 5 | 5 |  |  |  |  |  |  |  |  |  |  |  |  |  |  |  |  |  |  |  |
|  | WY | 13 | 11 |  |  |  |  | 2 |  |  |  |  |  |  |  |  |  |  |  |  |  |  |
|  | GY | 15 | 15 |  |  |  |  |  |  |  |  |  |  |  |  |  |  |  |  |  |  |  |
|  | JY | 14 | 13 |  |  |  |  | 1 |  |  |  |  |  |  |  |  |  |  |  |  |  |  |
|  | BX | 15 | 14 |  |  |  |  | 1 |  |  |  |  |  |  |  |  |  |  |  |  |  |  |
|  | KD | 23 | 6 |  |  |  |  | 11 |  |  | 5 | 1 |  |  |  |  |  |  |  |  |  |  |
|  | CY | 23 |  |  |  |  |  | 16 |  |  | 6 |  | 1 |  |  |  |  |  |  |  |  |  |
|  | ML | 15 |  |  |  |  |  | 15 |  |  |  |  |  |  |  |  |  |  |  |  |  |  |
|  | LZ | 17 |  |  |  |  |  | 17 |  |  |  |  |  |  |  |  |  |  |  |  |  |  |
|  | BM | 14 |  |  |  |  |  | 14 |  |  |  |  |  |  |  |  |  |  |  |  |  |  |
|  | GS | 15 |  |  |  |  |  | 15 |  |  |  |  |  |  |  |  |  |  |  |  |  |  |
|  | YH | 21 |  |  |  |  |  | 3 |  |  | 18 |  |  |  |  |  |  |  |  |  |  |  |
|  | LS | 15 |  |  |  |  |  |  |  |  | 15 |  |  |  |  |  |  |  |  |  |  |  |
|  | TC | 15 |  |  |  |  |  |  |  |  | 15 |  |  |  |  |  |  |  |  |  |  |  |
|  | CS | 15 |  |  |  |  |  |  |  |  | 15 |  |  |  |  |  |  |  |  |  |  |  |
|  | MY | 15 |  |  |  |  |  |  |  |  | 15 |  |  |  |  |  |  |  |  |  |  |  |
|  | SM | 16 |  |  |  |  |  |  |  |  | 16 |  |  |  |  |  |  |  |  |  |  |  |
|  | XY | 20 |  |  |  |  |  |  |  |  | 19 |  |  | 1 |  |  |  |  |  |  |  |  |
|  | CML | 15 |  |  |  |  |  | 7 |  |  | 8 |  |  |  |  |  |  |  |  |  |  |  |
|  | DQ | 15 |  |  |  |  |  | 13 |  |  |  |  |  |  | 2 |  |  |  |  |  |  |  |
| *P. armandii* var. *mastersiana* | DTS | 9 |  |  |  |  |  |  |  |  |  |  |  |  |  | 9 |  |  |  |  |  |  |
|  | YS | 7 |  |  |  |  |  |  |  |  |  |  |  |  |  | 7 |  |  |  |  |  |  |
| *P. wallichiana* | JL | 5 |  |  |  |  |  |  |  |  |  |  |  |  |  |  | 5 |  |  |  |  |  |
|  | YD | 5 |  |  |  |  |  |  |  |  |  |  |  |  |  |  | 2 | 3 |  |  |  |  |
|  | PAK2 | 5 |  |  |  |  |  |  |  |  |  |  |  |  |  |  | 5 |  |  |  |  |  |
|  | PAK7 | 5 |  |  |  |  |  |  |  |  |  |  |  |  |  |  | 5 |  |  |  |  |  |
| *P. kwangtungensis* | LB | 3 |  |  |  |  |  |  |  |  | 3 |  |  |  |  |  |  |  |  |  |  |  |
|  | YZ | 2 |  |  |  |  |  |  |  |  | 2 |  |  |  |  |  |  |  |  |  |  |  |
| *P. fenzeliana* | WM | 2 |  |  |  |  |  | 2 |  |  |  |  |  |  |  |  |  |  |  |  |  |  |
|  | HI | 2 |  |  |  |  |  |  |  |  | 2 |  |  |  |  |  |  |  |  |  |  |  |
|  | JFS | 6 |  |  |  |  |  | 3 |  |  |  |  |  |  |  |  |  |  | 1 | 2 |  |  |
| *P. bhutanica* | Bhu | 5 |  |  |  |  |  | 5 |  |  |  |  |  |  |  |  |  |  |  |  |  |  |
| *P. dabeshanensis* | YX | 2 |  |  |  |  |  | 2 |  |  |  |  |  |  |  |  |  |  |  |  |  |  |
|  | JZ | 1 |  |  |  |  |  | 1 |  |  |  |  |  |  |  |  |  |  |  |  |  |  |
|  | THC | 1 |  |  |  |  |  | 1 |  |  |  |  |  |  |  |  |  |  |  |  |  |  |
| *P. wangii* | MLP | 5 |  |  |  |  |  | 4 |  |  | 1 |  |  |  |  |  |  |  |  |  |  |  |
| *P. pumila* | WTE | 5 |  |  |  |  |  |  |  |  |  |  |  |  |  | 5 |  |  |  |  |  |  |
| *P. sibirica* | KNS | 5 |  |  |  |  |  |  |  |  |  |  |  |  |  | 5 |  |  |  |  |  |  |
| *P. morrisonicola* | NT | 1 |  |  |  |  |  |  |  |  |  |  |  |  |  | 1 |  |  |  |  |  |  |
|  | ML | 1 |  |  |  |  |  |  |  |  |  |  |  |  |  | 1 |  |  |  |  |  |  |
| *P. koraiensis* | CBS | 4 |  |  |  |  |  |  |  |  |  |  |  |  |  |  |  |  |  |  | 2 | 2 |
