# Supplementary material for: Phylogeography of Pinus armandii and Its Relatives: Heterogeneous Contributions of Geography and Climate Changes to the Genetic Differentiation and Diversification of Chinese White Pines
Source: PLoS One. 2014 Jan 21;9(1):e85920. doi: 10.1371/journal.pone.0085920 (PMC3897548; doi:10.1371/journal.pone.0085920)
Supplement: Table S3 — Mitotype distribution in each population of Pinus armandii and the other white pines in this study. (DOC) [file pone.0085920.s004.doc]

Table S3 Mitotype distribution in each population of *P*. *armandii* and the other white pines in this study.

| Species | Pop | n | Mitotype | | | | | | | | | | | | | | | | | | | | | | | | | |
| --- | --- | --- | --- | --- | --- | --- | --- | --- | --- | --- | --- | --- | --- | --- | --- | --- | --- | --- | --- | --- | --- | --- | --- | --- | --- | --- | --- | --- |
|  |  |  | M1 | M2 | M3 | M4 | M5 | M6 | M7 | M8 | M9 | M10 | M11 | M12 | M13 | M14 | M15 | M16 | M17 | M18 | M19 | M20 | M21 | M22 | M23 | M24 | M25 | M26 |
| *P*. *armandii* | DB | 3 | 3 |  |  |  |  |  |  |  |  |  |  |  |  |  |  |  |  |  |  |  |  |  |  |  |  |  |
|  | ZQ | 4 | 4 |  |  |  |  |  |  |  |  |  |  |  |  |  |  |  |  |  |  |  |  |  |  |  |  |  |
|  | WX | 17 | 14 | 2 | 1 |  |  |  |  |  |  |  |  |  |  |  |  |  |  |  |  |  |  |  |  |  |  |  |
|  | MX | 23 | 3 | 3 |  | 17 |  |  |  |  |  |  |  |  |  |  |  |  |  |  |  |  |  |  |  |  |  |  |
|  | LB | 5 | 4 | 1 |  |  |  |  |  |  |  |  |  |  |  |  |  |  |  |  |  |  |  |  |  |  |  |  |
|  | NS | 6 | 5 |  |  | 1 |  |  |  |  |  |  |  |  |  |  |  |  |  |  |  |  |  |  |  |  |  |  |
|  | XS | 17 |  |  |  | 1 | 11 | 2 | 1 | 2 |  |  |  |  |  |  |  |  |  |  |  |  |  |  |  |  |  |  |
|  | SNJ | 6 |  |  |  |  |  | 1 |  |  | 5 |  |  |  |  |  |  |  |  |  |  |  |  |  |  |  |  |  |
|  | HN | 5 |  |  | 5 |  |  |  |  |  |  |  |  |  |  |  |  |  |  |  |  |  |  |  |  |  |  |  |
|  | WY | 11 | 8 | 3 |  |  |  |  |  |  |  |  |  |  |  |  |  |  |  |  |  |  |  |  |  |  |  |  |
|  | GY | 15 | 11 | 4 |  |  |  |  |  |  |  |  |  |  |  |  |  |  |  |  |  |  |  |  |  |  |  |  |
|  | JY | 14 | 3 | 11 |  |  |  |  |  |  |  |  |  |  |  |  |  |  |  |  |  |  |  |  |  |  |  |  |
|  | BX | 14 | 13 |  |  |  |  |  |  |  |  | 1 |  |  |  |  |  |  |  |  |  |  |  |  |  |  |  |  |
|  | KD | 23 |  |  | 3 |  |  |  |  |  |  |  | 2 |  |  |  |  |  |  |  |  |  |  |  |  |  |  |  |
|  | CY | 23 |  |  |  |  |  |  |  |  |  |  |  | 23 |  |  |  |  |  |  |  |  |  |  |  |  |  |  |
|  | ML | 15 |  |  |  |  |  |  |  |  |  |  |  |  | 15 |  |  |  |  |  |  |  |  |  |  |  |  |  |
|  | LZ | 17 |  |  |  |  |  |  |  |  |  |  |  |  | 16 | 1 |  |  |  |  |  |  |  |  |  |  |  |  |
|  | BM | 14 |  |  |  |  |  |  |  |  |  |  |  |  | 14 |  |  |  |  |  |  |  |  |  |  |  |  |  |
|  | GS | 15 |  |  |  |  |  |  |  |  |  |  |  | 14 |  |  | 1 |  |  |  |  |  |  |  |  |  |  |  |
|  | YH | 21 |  |  |  | 9 |  |  |  |  |  |  | 12 |  |  |  |  |  |  |  |  |  |  |  |  |  |  |  |
|  | LS | 15 |  |  |  |  |  |  |  |  |  |  |  |  | 15 |  |  |  |  |  |  |  |  |  |  |  |  |  |
|  | TC | 15 |  |  |  |  |  |  |  |  |  |  | 15 |  |  |  |  |  |  |  |  |  |  |  |  |  |  |  |
|  | CS | 15 |  |  |  | 4 |  |  |  |  |  |  | 1 |  |  |  |  | 1 |  |  |  |  |  |  |  |  |  |  |
|  | MY | 15 |  |  |  | 1 |  |  |  |  |  |  |  |  |  |  |  | 5 |  |  |  |  |  |  |  |  |  |  |
|  | SM | 16 |  |  |  | 11 |  |  |  |  |  |  |  |  |  |  |  | 5 |  |  |  |  |  |  |  |  |  |  |
|  | XY | 20 |  |  |  | 7 |  |  |  |  |  |  |  |  |  |  |  | 13 |  |  |  |  |  |  |  |  |  |  |
|  | CML | 15 |  |  |  |  |  |  |  |  |  |  | 15 |  |  |  |  |  |  |  |  |  |  |  |  |  |  |  |
|  | DQ | 15 |  |  |  |  |  |  |  |  |  |  |  |  |  |  |  |  | 15 |  |  |  |  |  |  |  |  |  |
| *P*. *armandii* var. *masteriana* | YS | 1 |  |  |  |  |  |  |  |  |  |  |  |  |  |  |  |  |  | 1 |  |  |  |  |  |  |  |  |
| *P*. *wallichiana* | JL | 5 |  |  |  |  |  |  |  |  |  |  |  |  |  |  |  |  |  |  | 3 | 2 |  |  |  |  |  |  |
|  | YD | 5 |  |  |  |  |  |  |  |  |  |  |  |  |  |  |  |  |  |  |  |  | 5 |  |  |  |  |  |
|  | PAK2 | 5 |  |  |  |  |  |  |  |  |  |  |  |  |  |  |  |  |  |  | 5 |  |  |  |  |  |  |  |
|  | PAK7 | 5 |  |  |  |  |  |  |  |  |  |  |  |  |  |  |  |  |  |  | 5 |  |  |  |  |  |  |  |
| *P. kwangtungensis* | LB | 3 |  |  |  |  |  |  |  |  |  |  |  |  |  |  |  | 3 |  |  |  |  |  |  |  |  |  |  |
|  | YZ | 2 |  |  |  |  |  |  |  |  |  |  |  | 2 |  |  |  |  |  |  |  |  |  |  |  |  |  |  |
| *P. fenzeliana* | WM | 2 |  |  |  |  |  |  |  |  |  |  |  |  |  |  |  |  |  |  |  |  |  | 2 |  |  |  |  |
|  | HI | 2 |  |  |  |  |  |  |  |  |  |  |  | 2 |  |  |  |  |  |  |  |  |  |  |  |  |  |  |
|  | JFS | 6 |  |  |  | 6 |  |  |  |  |  |  |  |  |  |  |  |  |  |  |  |  |  |  |  |  |  |  |
| *P. bhutanica* | Bhu | 5 |  |  |  |  |  |  |  |  |  |  |  | 5 |  |  |  |  |  |  |  |  |  |  |  |  |  |  |
| *P. dabeshanensis* | YX | 2 |  |  |  |  |  |  |  |  |  |  | 2 |  |  |  |  |  |  |  |  |  |  |  |  |  |  |  |
|  | JZ | 1 |  |  |  |  |  |  |  |  |  |  | 1 |  |  |  |  |  |  |  |  |  |  |  |  |  |  |  |
|  | THC | 1 |  |  |  |  |  |  |  |  |  |  | 1 |  |  |  |  |  |  |  |  |  |  |  |  |  |  |  |
| *P. wangii* | MLP | 5 |  |  |  |  |  |  |  |  |  |  |  |  |  |  |  |  |  |  |  |  |  |  | 5 |  |  |  |
| *P. pumila* | WTE | 5 |  |  |  |  |  |  |  |  |  |  |  |  |  |  |  |  |  |  |  |  |  |  |  | 5 |  |  |
| *P. sibirica* | KNS | 5 |  |  |  |  |  |  |  |  |  |  |  |  |  |  |  |  |  |  |  |  |  |  |  |  | 5 |  |
| *P. morrisonicola* | NT | 1 |  |  |  |  |  |  |  |  |  |  |  |  |  |  |  |  |  | 1 |  |  |  |  |  |  |  |  |
|  | ML | 1 |  |  |  |  |  |  |  |  |  |  |  |  |  |  |  |  |  | 1 |  |  |  |  |  |  |  |  |
| *P. koraiensis* | CBS | 4 |  |  |  |  |  |  |  |  |  |  |  |  |  |  |  |  |  |  |  |  |  |  |  |  |  | 4 |
